# Poly[μ-4,4′-bipyridine-κ^2^ *N*:*N*′-μ-thio­cyanato-κ^2^ *N*:*S*-copper(I)]

**DOI:** 10.1107/S1600536808033175

**Published:** 2008-10-18

**Authors:** Mario Wriedt, Sina Sellmer, Christian Näther

**Affiliations:** a Institut für Anorganische Chemie, Christian-Albrechts-Universität Kiel, Max-Eyth-Strasse 2, D-24118 Kiel, Germany

## Abstract

In the crystal structure of the title compound, [Cu(NCS)(C_10_H_8_N_2_)]_*n*_, the Cu^I^ atom is coordinated by two N atoms from two symmetry-related 4,4′-bipyridine (bipy) ligands and one N and one S atom from two symmetry-related thio­cyanate ligands in a distorted tetra­hedral environment. The thio­cyanate ligands bridge the Cu^I^ atoms into a zigzag [CuSCN]_*n*_ chain running parallel to the *a* axis. These chains are further connected through two bipy ligands that bridge the Cu^I^ centers to generate a two-dimensional brick-like network. The pyridyl planes of the ligands exhibit a dihedral angle of 37.35 (12)°.

## Related literature

For related structures, see: Goher & Mautner (1999 ▶); Teichert & Sheldrick (1999 ▶); Wang *et al.* (1999 ▶). For related chemistry, see: Bhosekar *et al.* (2007 ▶); Healy *et al.* (1984 ▶); Näther & Greve (2003 ▶); Näther & Jess (2001 ▶, 2006 ▶); Näther *et al.* (2002 ▶); Näther, Greve & Jess (2003 ▶); Näther, Wriedt & Jess (2003 ▶).
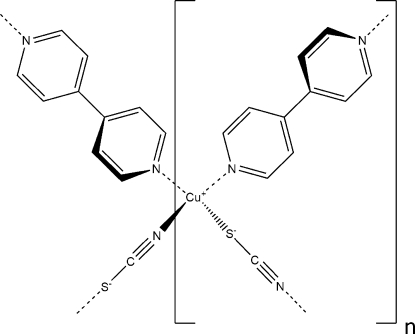


## Experimental

### 

#### Crystal data

[Cu(NCS)(C_10_H_8_N_2_)]
*M* *_r_* = 277.80
Orthorhombic, 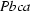

*a* = 11.4340 (4) Å
*b* = 12.2530 (5) Å
*c* = 15.3806 (6) Å
*V* = 2154.83 (14) Å^3^
*Z* = 8
Mo *K*α radiation
μ = 2.19 mm^−1^
*T* = 170 (2) K
0.12 × 0.08 × 0.05 mm

#### Data collection

Stoe IPDS-II diffractometer
Absorption correction: numerical (*X-SHAPE* and *X-RED32*; Stoe & Cie, 2008 ▶) *T*_min_ = 0.817, *T*_max_ = 0.901
23916 measured reflections
2915 independent reflections
2567 reflections with *I* > 2σ(*I*)
*R*_int_ = 0.040

#### Refinement

*R*[*F* ^2^ > 2σ(*F* ^2^)] = 0.047
*wR*(*F* ^2^) = 0.090
*S* = 1.24
2915 reflections
146 parameters
H-atom parameters constrained
Δρ_max_ = 0.32 e Å^−3^
Δρ_min_ = −0.43 e Å^−3^

### 

Data collection: *X-AREA* (Stoe & Cie, 2008 ▶); cell refinement: *X-AREA*; data reduction: *X-AREA*; program(s) used to solve structure: *SHELXS97* (Sheldrick, 2008 ▶); program(s) used to refine structure: *SHELXL97* (Sheldrick, 2008 ▶); molecular graphics: *XP* in *SHELXTL* (Sheldrick, 2008 ▶); software used to prepare material for publication: *XCIF* in *SHELXTL*.

## Supplementary Material

Crystal structure: contains datablocks I, global. DOI: 10.1107/S1600536808033175/bt2809sup1.cif

Structure factors: contains datablocks I. DOI: 10.1107/S1600536808033175/bt2809Isup2.hkl

Additional supplementary materials:  crystallographic information; 3D view; checkCIF report

## Figures and Tables

**Table:** 

Cu1—N11	1.966 (2)
Cu1—N1	2.080 (2)
Cu1—N2^i^	2.122 (2)
Cu1—S11^ii^	2.2755 (8)
N11—C11	1.151 (3)
C11—S11	1.651 (3)

**Table:** 

N11—Cu1—N1	111.31 (9)
N11—Cu1—N2^i^	101.07 (9)
N1—Cu1—N2^i^	97.36 (9)
N11—Cu1—S11^ii^	115.22 (7)
N1—Cu1—S11^ii^	111.96 (6)
N2^i^—Cu1—S11^ii^	118.21 (6)

Symmetry codes: (i) 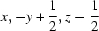; (ii) 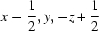.

**Fig. 1.**
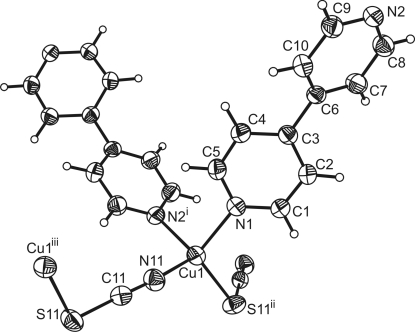
Crystal structure of the title compund with labelling and displacement ellipsoids drawn at the 50% probability level. [Symmetry codes: i = x, -y + 1/2, z - 1/2; ii = x - 1/2, y, -z + 1/2; iii = x + 1/2, y, -z + 1/2.]

**Fig. 2.**
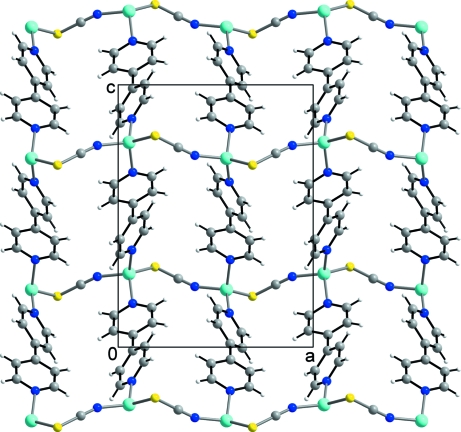
Crystal structure of the title compound with view along the b axis.

**Fig. 3.**
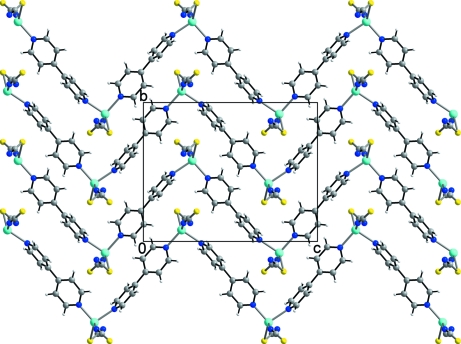
Crystal structure of the title compound with view along the a axis.

## supplementary crystallographic information

### Comment

In our ongoing investigation on the synthesis, structures and properties of new coordination polymers based on metal halides as well as pseudohalides and N-donor ligands, we have startet systematic investigation on their thermal behavior because we have demonstrated that new ligand deficient coordination polymers can be conveniently prepared by thermal decompisition of suitable ligand rich precursor compounds (Näther, Wriedt & Jeß, 2003; Näther & Jeß, 2006; Bhosekar *et al.* 2007). If the ligand rich precursor compounds contain besides the N-donor ligands paramagnetic metal atoms and small magnetically active ligands like SCN^-^, ligand deficient compounds with briding SCN^-^ ligands are obtained, which show cooperative magnetic phenomena at lower temperatures (Näther & Greve, 2003). During these investigations we have reacted copper(II)chloride and potassium thiocyanate with bipy. In this reaction the diamagnetic copper(I) title compound has been formed by accident.

The coordination properties of bipy enables a series of different coordination modes, because it can connect two different metal cations. In addition, typical Cu—S—C angles in CuSCN polymers are in the range of 100–106° (Healy *et al.* 1984) and this should enable the construction of stairlike single or double [Cu(SCN)] chains in 1:1 and 2:1 complexes, whose Cu atoms can then be connected by linear spacer ligands into sheets (Näther & Jeß, 2001; Näther *et al.* 2002; Näther, Greve & Jeß, 2003).

The 1:1 title compound [CuSCN(bipy)]_n_, whose structure (Fig. 1) represents a two-dimensional CuSCN coordination polymer, contains single [CuSCN] ribbons (Fig. 2) as a characteristic motif. Copper(i) thiocyanato compounds with pyrazine (Goher & Mautner, 1999), methylpyrazine (Teichert & Sheldrick, 1999) and 1,2-bis(4-pyridyl)ethane (Wang *et al.* 1999) as ligand show a similar topology. Within each layer the metal ions are bridged by two *µ*_2_(*N*,*N'*)-bipy ligands and two *µ*(*N*,*S*)-thiocyanato groups. Thus, each copper(i) atom is tetrahedrally coordinated. The angels arround the copper(i) atoms range between 97.36 (9) and 115.22 (7)° and the Cu—SCN and Cu—NCS distances amount to 2.2755 (8) and 1.966 (2) Å, respectively. The Cu—N_bipy_ distances ranges from 2.080 (2) to 2.122 (2) Å (Tab. 1). The layers can be described as formed by two types of perpendicular zigzag like chains crossing at the copper(i) centers. Chains of the first type run along the *c*-axis and have bipy as a bridging ligand, while the second type extend along the *a*-axis containing bridging thiocyanate ligands. The intralayer Cu···Cu distances are 5.7942 (2) and 11.2037 (3) Å for Cu—NCS—Cu and Cu—bipy—Cu, respectively. The packing of the crystal structure is achieved by stacking the two-dimensional layers along the *b*-axis in corrugated sheets (Fig. 3) with an interlayer stacking distance between the centroides of the sixmembered rings of 4.237 (2) Å.

### Experimental

CuCl_2_ and bipy was obtained from Alfa Aesar, KSCN and methanol was obtained from Fluka. 0.1 mmol (13.4 mg) CuCl_2_, 0.2 mmol (19.4 mg) KSCN, 0.6 mmol (93.7 mg) and 1 ml of methanol were transfered in a test-tube, which was closed and heated to 120 °C for three days. On cooling orange block-shaped single crystals of the title compound were obtained.

### Refinement

All H atoms were located in difference map but were positioned with idealized geometry and were refined isotropic with *U*_eq_(H) = 1.2 *U*_eq_(C) of the parent atom using a riding model with C—H = 0.95 Å.

### Crystal data

**Table:**

[Cu(NCS)(C_10_H_8_N_2_)]	*D*_x_ = 1.713 Mg m^−^^3^
*M**_r_* = 277.80	Mo *K*α radiation, λ = 0.71073 Å
Orthorhombic, *P**b**c**a*	Cell parameters from 23129 reflections
*a* = 11.4340 (4) Å	θ = 1.7–29.7°
*b* = 12.2530 (5) Å	µ = 2.19 mm^−^^1^
*c* = 15.3806 (6) Å	*T* = 170 K
*V* = 2154.83 (14) Å^3^	Block, orange
*Z* = 8	0.12 × 0.08 × 0.05 mm
*F*(000) = 1120	

### Data collection

**Table:**

Stoe IPDS-II diffractometer	2915 independent reflections
Radiation source: fine-focus sealed tube	2567 reflections with *I* > 2σ(*I*)
graphite	*R*_int_ = 0.040
ω scans	θ_max_ = 29.3°, θ_min_ = 2.7°
Absorption correction: numerical (X-SHAPE and X-RED32; Stoe & Cie, 2008)	*h* = −15→15
*T*_min_ = 0.817, *T*_max_ = 0.901	*k* = −16→16
23916 measured reflections	*l* = −21→20

### Refinement

**Table:**

Refinement on *F*^2^	Secondary atom site location: difference Fourier map
Least-squares matrix: full	Hydrogen site location: inferred from neighbouring sites
*R*[*F*^2^ > 2σ(*F*^2^)] = 0.047	H-atom parameters constrained
*wR*(*F*^2^) = 0.090	*w* = 1/[σ^2^(*F*_o_^2^) + (0.0317*P*)^2^ + 1.4342*P*] where *P* = (*F*_o_^2^ + 2*F*_c_^2^)/3
*S* = 1.24	(Δ/σ)_max_ = 0.001
2915 reflections	Δρ_max_ = 0.32 e Å^−^^3^
146 parameters	Δρ_min_ = −0.43 e Å^−^^3^
0 restraints	Extinction correction: SHELXL97 (Sheldrick, 2008), Fc^*^=kFc[1+0.001xFc^2^λ^3^/sin(2θ)]^-1/4^
Primary atom site location: structure-invariant direct methods	Extinction coefficient: 0.0015 (3)

### Special details

**Table:**

Geometry. All e.s.d.'s (except the e.s.d. in the dihedral angle between two l.s. planes) are estimated using the full covariance matrix. The cell e.s.d.'s are taken into account individually in the estimation of e.s.d.'s in distances, angles and torsion angles; correlations between e.s.d.'s in cell parameters are only used when they are defined by crystal symmetry. An approximate (isotropic) treatment of cell e.s.d.'s is used for estimating e.s.d.'s involving l.s. planes.
Refinement. Refinement of *F*^2^ against ALL reflections. The weighted *R*-factor *wR* and goodness of fit *S* are based on *F*^2^, conventional *R*-factors *R* are based on *F*, with *F* set to zero for negative *F*^2^. The threshold expression of *F*^2^ > σ(*F*^2^) is used only for calculating *R*-factors(gt) *etc*. and is not relevant to the choice of reflections for refinement. *R*-factors based on *F*^2^ are statistically about twice as large as those based on *F*, and *R*- factors based on ALL data will be even larger.

### Fractional atomic coordinates and isotropic or equivalent isotropic displacement parameters (Å^2^)

**Table:**

	*x*	*y*	*z*	*U*_iso_*/*U*_eq_	
Cu1	0.44386 (3)	0.58247 (3)	0.28065 (2)	0.03676 (11)	
N1	0.42996 (19)	0.45592 (18)	0.37017 (14)	0.0353 (5)	
N2	0.4249 (2)	0.01317 (17)	0.66651 (14)	0.0361 (5)	
C1	0.3680 (2)	0.4601 (2)	0.44384 (17)	0.0385 (6)	
H1	0.3252	0.5247	0.4561	0.046*	
C2	0.3631 (2)	0.3757 (2)	0.50299 (17)	0.0388 (6)	
H2	0.3176	0.3825	0.5544	0.047*	
C3	0.4252 (2)	0.2806 (2)	0.48664 (16)	0.0325 (5)	
C4	0.4902 (2)	0.2762 (2)	0.41074 (17)	0.0372 (5)	
H4	0.5350	0.2132	0.3972	0.045*	
C5	0.4892 (2)	0.3641 (2)	0.35506 (17)	0.0387 (6)	
H5	0.5332	0.3591	0.3028	0.046*	
C6	0.4236 (2)	0.18673 (19)	0.54777 (15)	0.0314 (5)	
C7	0.3241 (2)	0.1573 (2)	0.59312 (19)	0.0404 (6)	
H7	0.2532	0.1963	0.5845	0.048*	
C8	0.3280 (2)	0.0711 (2)	0.65107 (18)	0.0414 (6)	
H8	0.2585	0.0521	0.6813	0.050*	
C9	0.5207 (2)	0.0408 (2)	0.62150 (17)	0.0389 (6)	
H9	0.5902	−0.0001	0.6308	0.047*	
C10	0.5238 (2)	0.1255 (2)	0.56241 (17)	0.0375 (6)	
H10	0.5939	0.1417	0.5320	0.045*	
N11	0.6066 (2)	0.6321 (2)	0.26898 (16)	0.0419 (5)	
C11	0.6915 (2)	0.6652 (2)	0.23866 (16)	0.0342 (5)	
S11	0.81061 (6)	0.71667 (6)	0.19394 (5)	0.04387 (18)	

### Atomic displacement parameters (Å^2^)

**Table:**

	*U*^11^	*U*^22^	*U*^33^	*U*^12^	*U*^13^	*U*^23^
Cu1	0.03545 (17)	0.03621 (17)	0.03862 (17)	−0.00274 (13)	0.00331 (14)	0.00139 (13)
N1	0.0374 (11)	0.0341 (10)	0.0342 (11)	−0.0018 (9)	0.0027 (9)	0.0024 (9)
N2	0.0416 (12)	0.0339 (10)	0.0329 (11)	−0.0005 (9)	0.0020 (9)	0.0028 (8)
C1	0.0445 (15)	0.0336 (12)	0.0375 (13)	0.0026 (11)	0.0052 (11)	−0.0007 (11)
C2	0.0459 (15)	0.0372 (13)	0.0334 (12)	0.0005 (11)	0.0078 (11)	0.0011 (11)
C3	0.0352 (12)	0.0322 (11)	0.0302 (11)	−0.0046 (9)	−0.0020 (9)	0.0001 (9)
C4	0.0419 (14)	0.0338 (12)	0.0359 (13)	0.0039 (11)	0.0027 (11)	0.0004 (10)
C5	0.0430 (14)	0.0406 (13)	0.0323 (12)	0.0017 (11)	0.0053 (11)	0.0022 (11)
C6	0.0386 (13)	0.0275 (10)	0.0281 (11)	−0.0027 (9)	−0.0021 (9)	−0.0015 (9)
C7	0.0374 (14)	0.0366 (13)	0.0472 (15)	0.0009 (11)	0.0032 (11)	0.0052 (11)
C8	0.0385 (14)	0.0411 (14)	0.0446 (14)	−0.0020 (11)	0.0043 (11)	0.0049 (12)
C9	0.0397 (13)	0.0404 (13)	0.0366 (13)	0.0038 (11)	0.0004 (11)	0.0035 (11)
C10	0.0375 (13)	0.0418 (14)	0.0332 (12)	−0.0012 (11)	0.0027 (10)	0.0030 (11)
N11	0.0348 (12)	0.0455 (13)	0.0455 (13)	−0.0060 (10)	−0.0005 (10)	−0.0006 (10)
C11	0.0322 (12)	0.0330 (12)	0.0374 (13)	0.0021 (10)	−0.0057 (10)	−0.0006 (10)
S11	0.0321 (3)	0.0384 (3)	0.0611 (4)	−0.0008 (3)	0.0043 (3)	0.0118 (3)

### Geometric parameters (Å, °)

**Table:**

Cu1—N11	1.966 (2)	C4—C5	1.376 (4)
Cu1—N1	2.080 (2)	C4—H4	0.9500
Cu1—N2^i^	2.122 (2)	C5—H5	0.9500
Cu1—S11^ii^	2.2755 (8)	C6—C7	1.382 (4)
N1—C5	1.333 (3)	C6—C10	1.388 (4)
N1—C1	1.337 (3)	C7—C8	1.383 (4)
N2—C8	1.336 (4)	C7—H7	0.9500
N2—C9	1.339 (3)	C8—H8	0.9500
N2—Cu1^iii^	2.122 (2)	C9—C10	1.380 (4)
C1—C2	1.379 (4)	C9—H9	0.9500
C1—H1	0.9500	C10—H10	0.9500
C2—C3	1.388 (4)	N11—C11	1.151 (3)
C2—H2	0.9500	C11—S11	1.651 (3)
C3—C4	1.385 (4)	S11—Cu1^iv^	2.2755 (8)
C3—C6	1.485 (3)		
			
N11—Cu1—N1	111.31 (9)	C3—C4—H4	120.3
N11—Cu1—N2^i^	101.07 (9)	N1—C5—C4	123.8 (2)
N1—Cu1—N2^i^	97.36 (9)	N1—C5—H5	118.1
N11—Cu1—S11^ii^	115.22 (7)	C4—C5—H5	118.1
N1—Cu1—S11^ii^	111.96 (6)	C7—C6—C10	117.2 (2)
N2^i^—Cu1—S11^ii^	118.21 (6)	C7—C6—C3	122.1 (2)
C5—N1—C1	116.7 (2)	C10—C6—C3	120.7 (2)
C5—N1—Cu1	118.34 (17)	C6—C7—C8	119.8 (3)
C1—N1—Cu1	124.96 (18)	C6—C7—H7	120.1
C8—N2—C9	116.8 (2)	C8—C7—H7	120.1
C8—N2—Cu1^iii^	121.67 (18)	N2—C8—C7	123.2 (3)
C9—N2—Cu1^iii^	118.92 (18)	N2—C8—H8	118.4
N1—C1—C2	123.5 (3)	C7—C8—H8	118.4
N1—C1—H1	118.2	N2—C9—C10	123.5 (3)
C2—C1—H1	118.2	N2—C9—H9	118.3
C1—C2—C3	119.3 (2)	C10—C9—H9	118.3
C1—C2—H2	120.4	C9—C10—C6	119.5 (2)
C3—C2—H2	120.4	C9—C10—H10	120.3
C4—C3—C2	117.4 (2)	C6—C10—H10	120.3
C4—C3—C6	120.7 (2)	C11—N11—Cu1	160.8 (2)
C2—C3—C6	121.9 (2)	N11—C11—S11	177.9 (2)
C5—C4—C3	119.4 (2)	C11—S11—Cu1^iv^	101.83 (9)
C5—C4—H4	120.3		

Symmetry codes: (i) *x*, −*y*+1/2, *z*−1/2; (ii) *x*−1/2, *y*, −*z*+1/2; (iii) *x*, −*y*+1/2, *z*+1/2; (iv) *x*+1/2, *y*, −*z*+1/2.
